# Supplementary material for: LAMP2A-dependent chaperone-mediated autophagy enhances oxidative stress resistance in gastric cancer cells through selective degradation of accumulated oxidized DJ-1
Source: PLoS One. 2026 May 15;21(5):e0331823. doi: 10.1371/journal.pone.0331823 (PMC13178873; doi:10.1371/journal.pone.0331823)

Figure1D

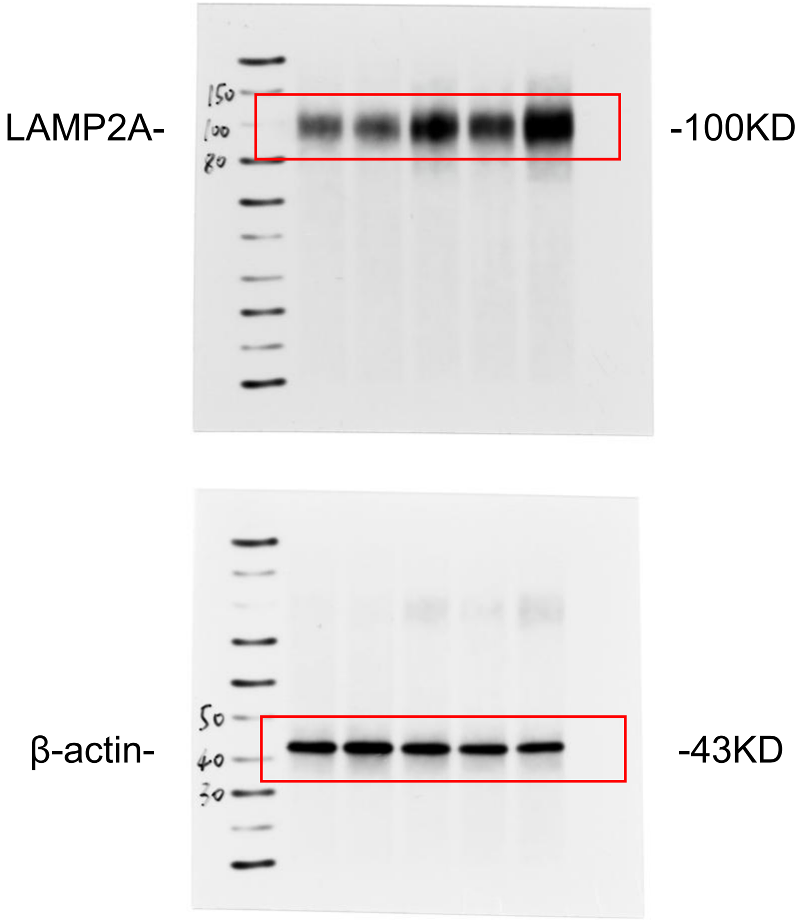

Figure2

AGS

LAMP2A- 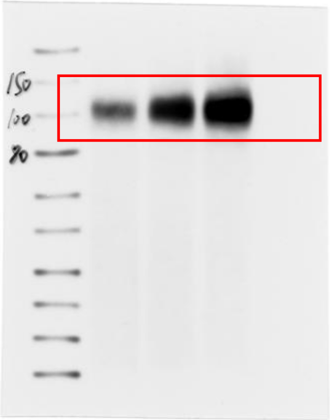 -100KD

β-actin- 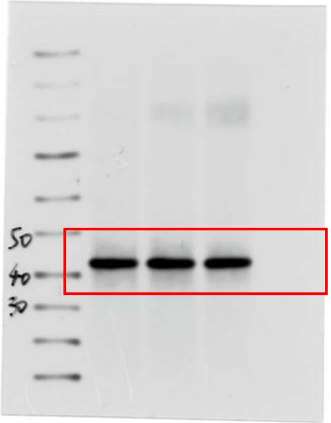 -43KD

MKN45

LAMP2A- 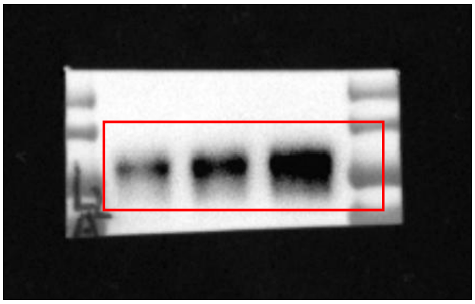 -100KD

β-actin- 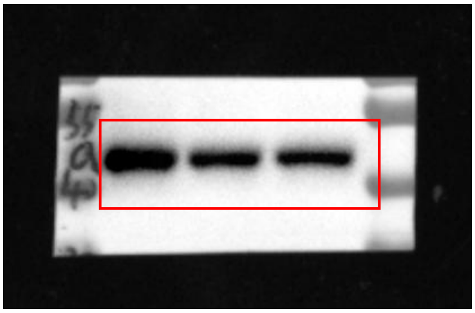 -43KD

Figure2

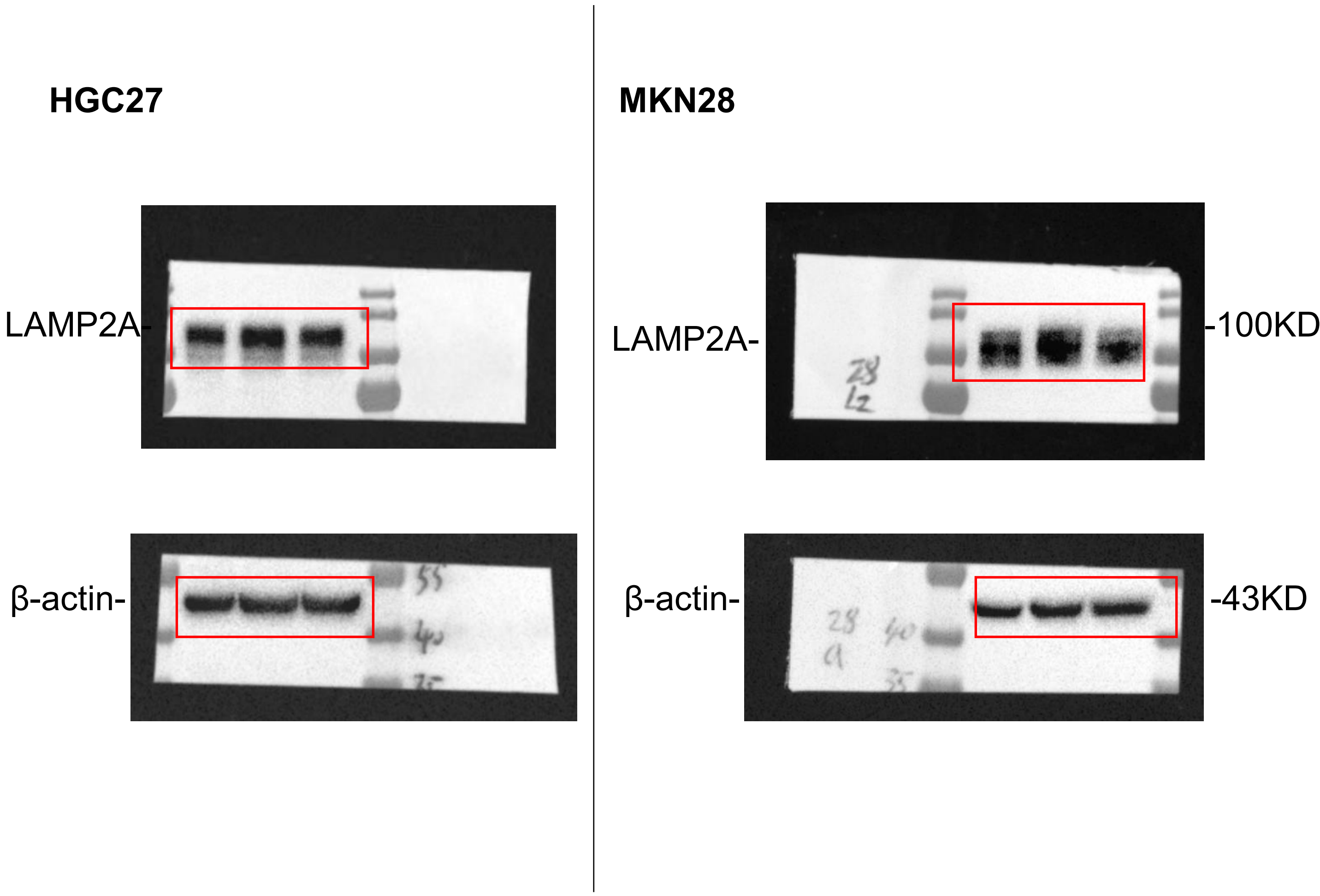

Figure3B

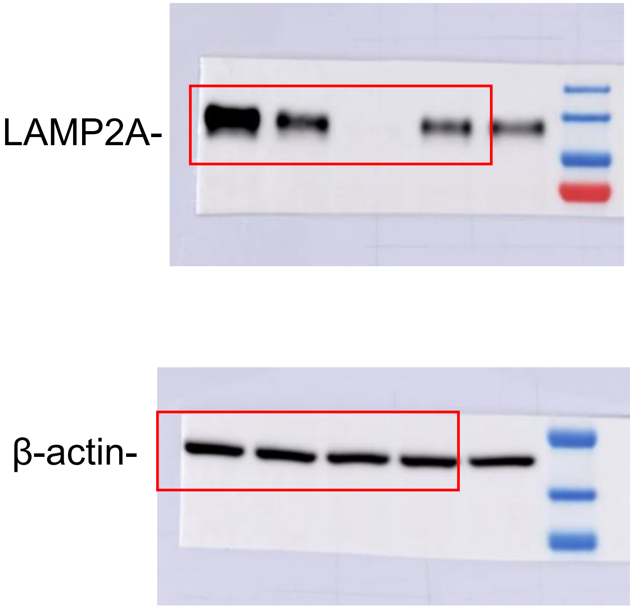

Figure3D

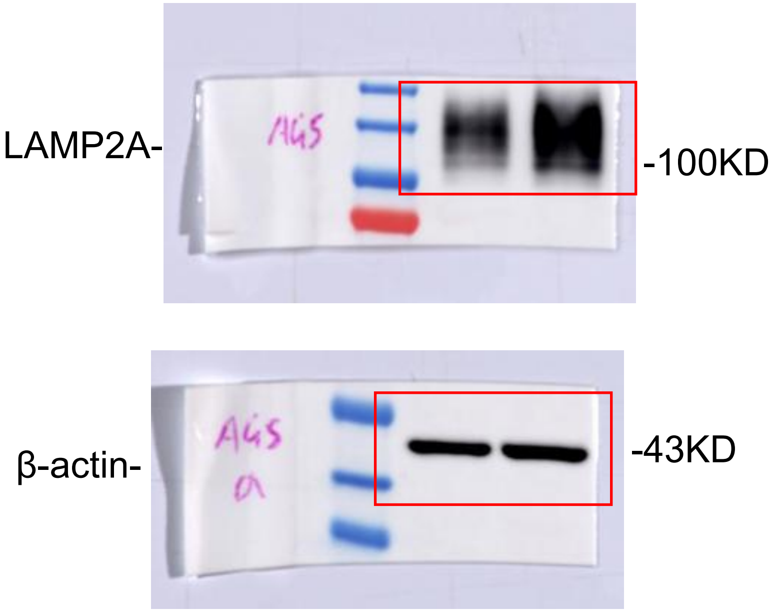

Figure5B

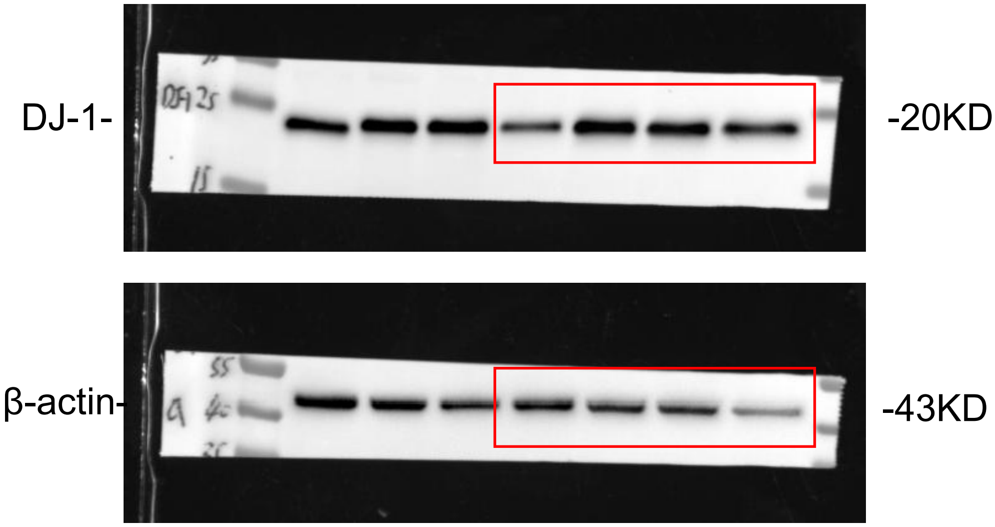

Figure5C

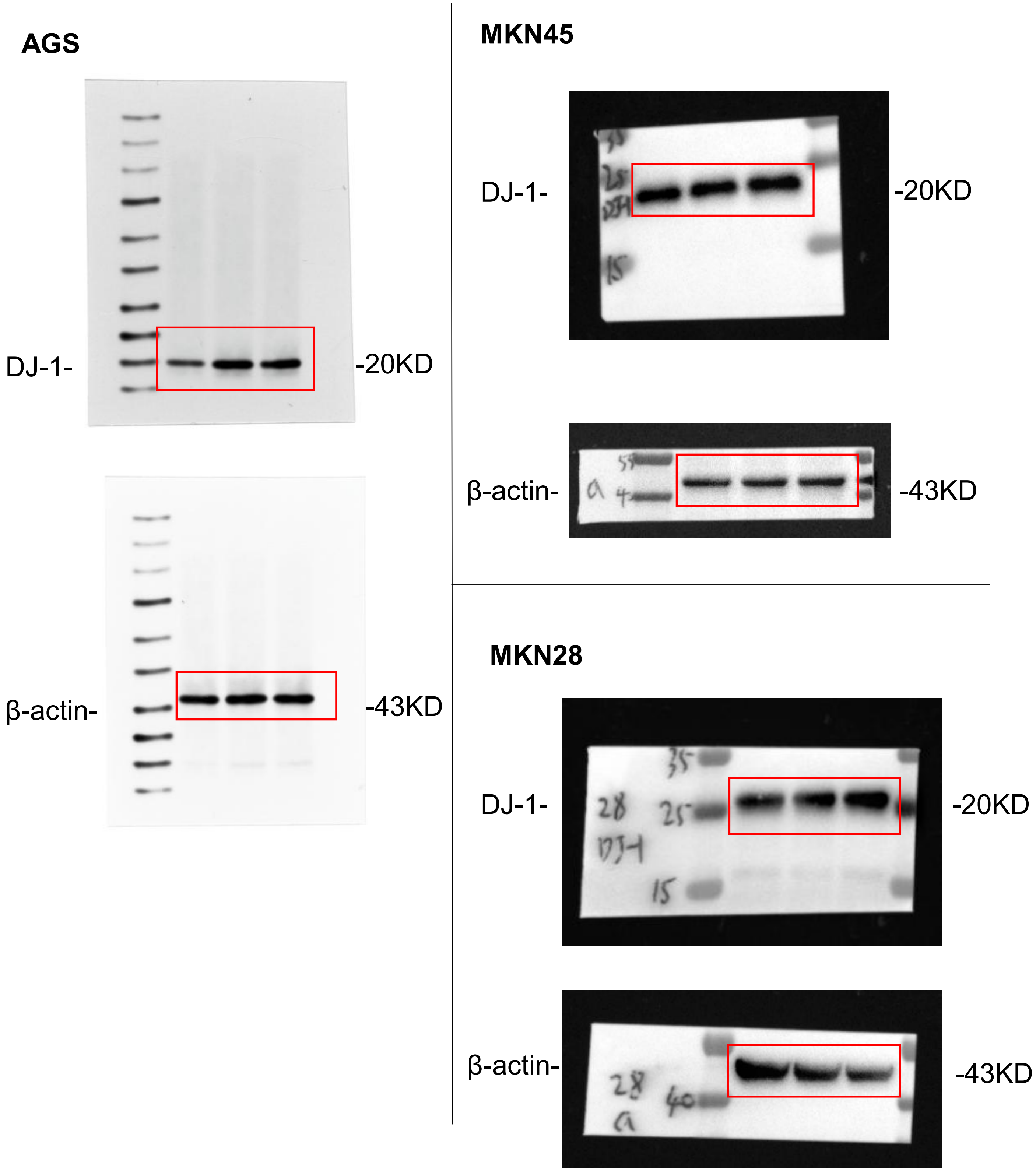

AGS
DJ-1-
-20KD
β-actin-
-43KD
MKN45
DJ-1-
-20KD
β-actin-
-43KD
MKN28
DJ-1-
-20KD
β-actin-
-43KD

Figure5D

LAMP2A- -100KD

DJ-1- -20KD

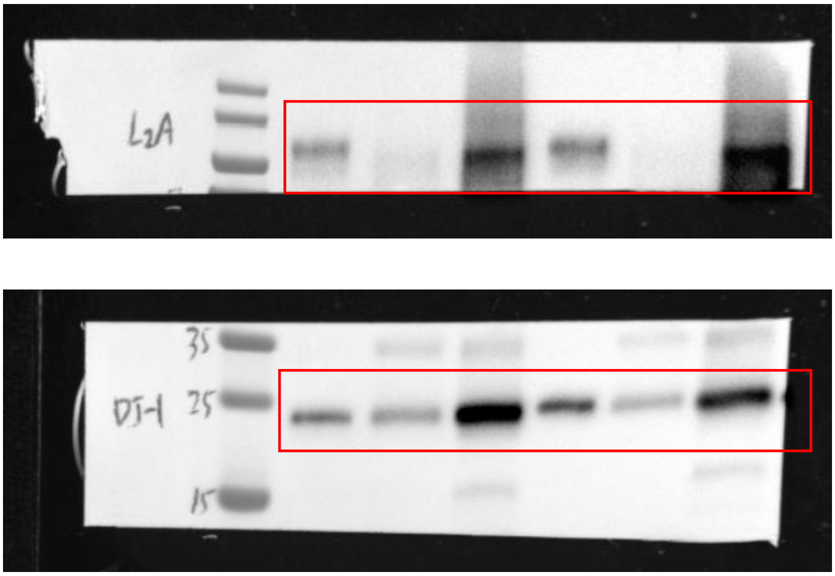

Figure6A

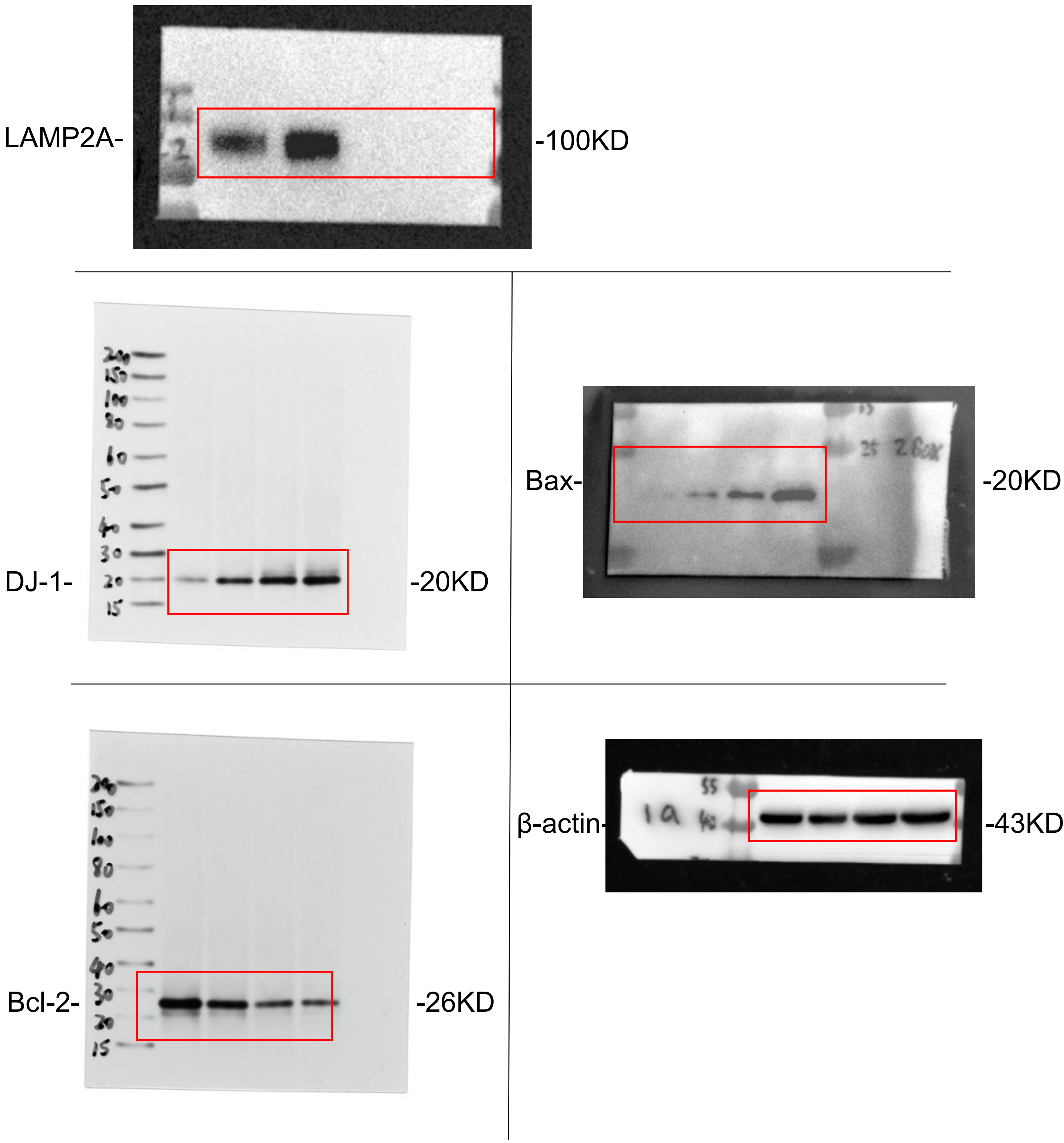

Figure6B

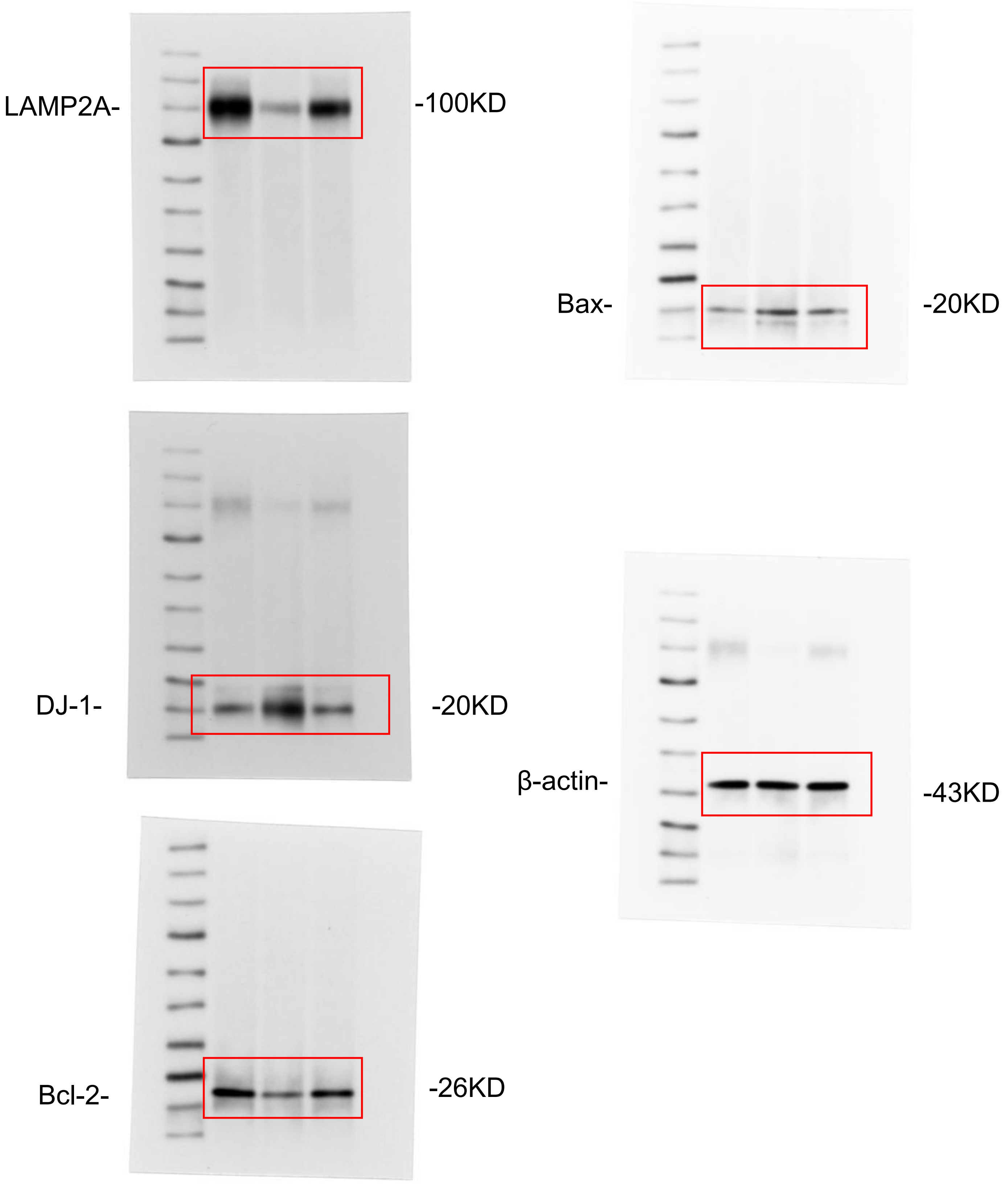

FigureS3

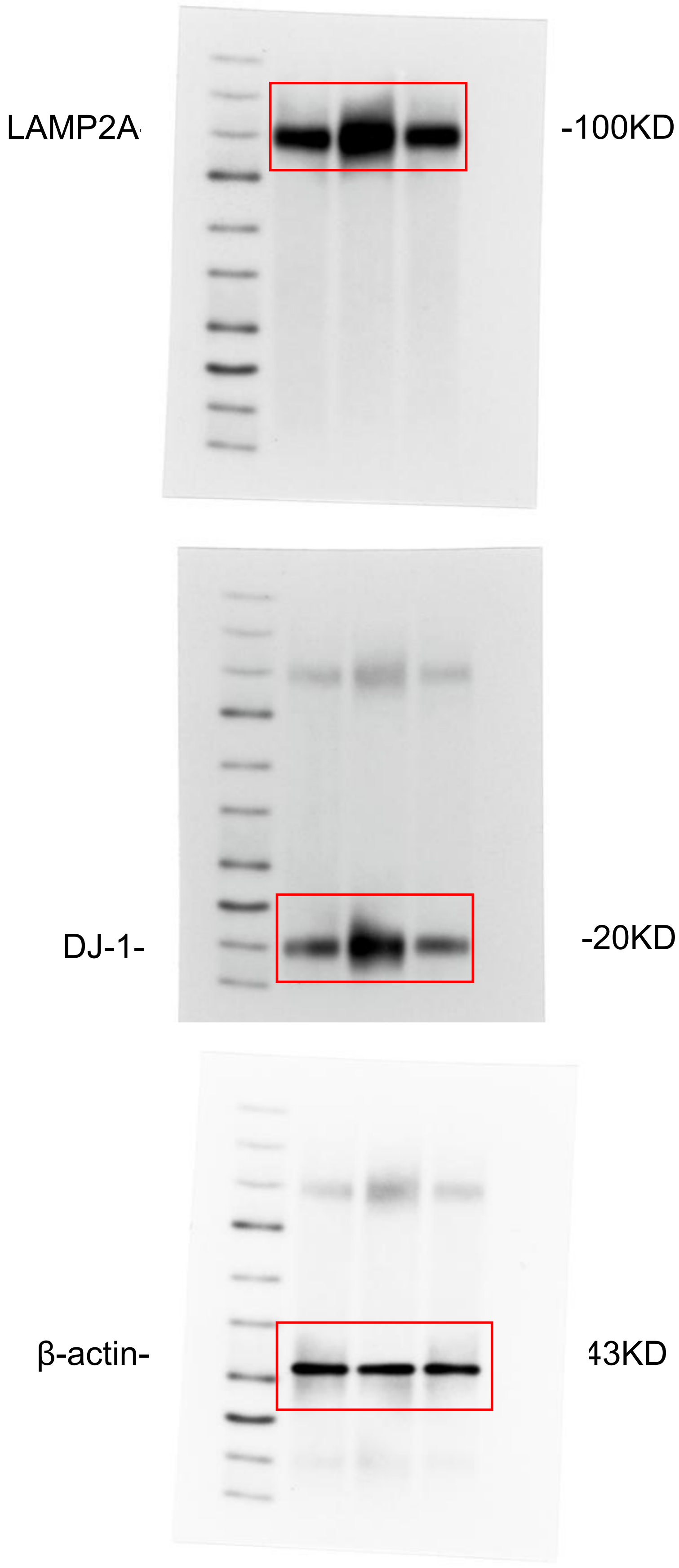

Supplement: S1 Raw Western Blot Images — (PDF) [file pone.0331823.s001.pdf]
