# Supplementary figures and images for: LAMP2A-dependent chaperone-mediated autophagy enhances oxidative stress resistance in gastric cancer cells through selective degradation of accumulated oxidized DJ-1
Source: PLoS One. 2026 May 15;21(5):e0331823. doi: 10.1371/journal.pone.0331823 (PMC13178873; doi:10.1371/journal.pone.0331823)

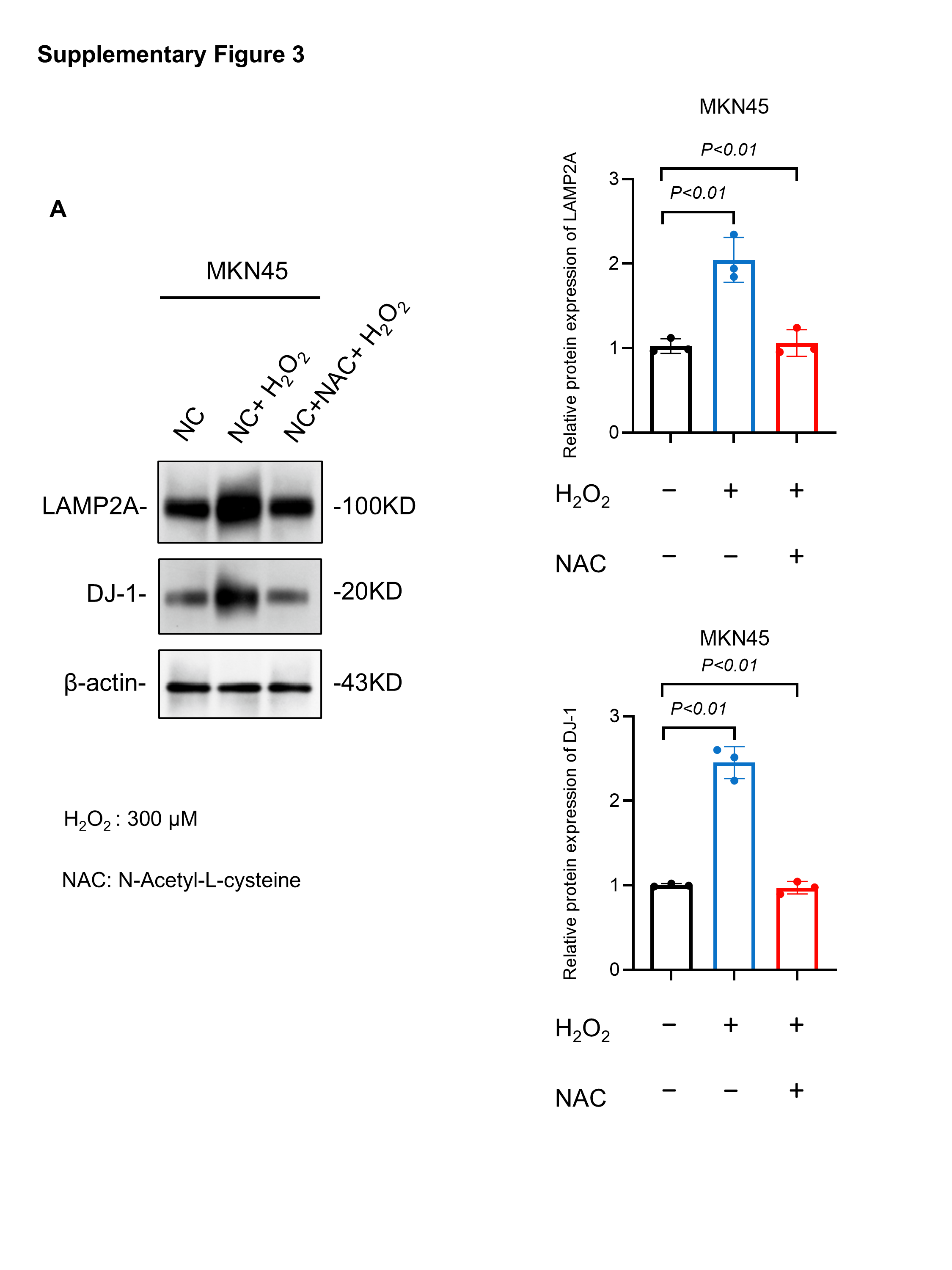

Supplement: S3 Fig — (A)Western blot analysis shows protein levels of LAMP2A and DJ-1 in gastric cancer cells pretreated with or without NAC (5 mM, 2 h) followed by exposure to H₂O₂ (300 μM, 24 h). Data are presented as mean±SD of three independent experiments. ns, no significance, *P < 0.05. **P < 0.01. (TIF) [file pone.0331823.s003.tif]

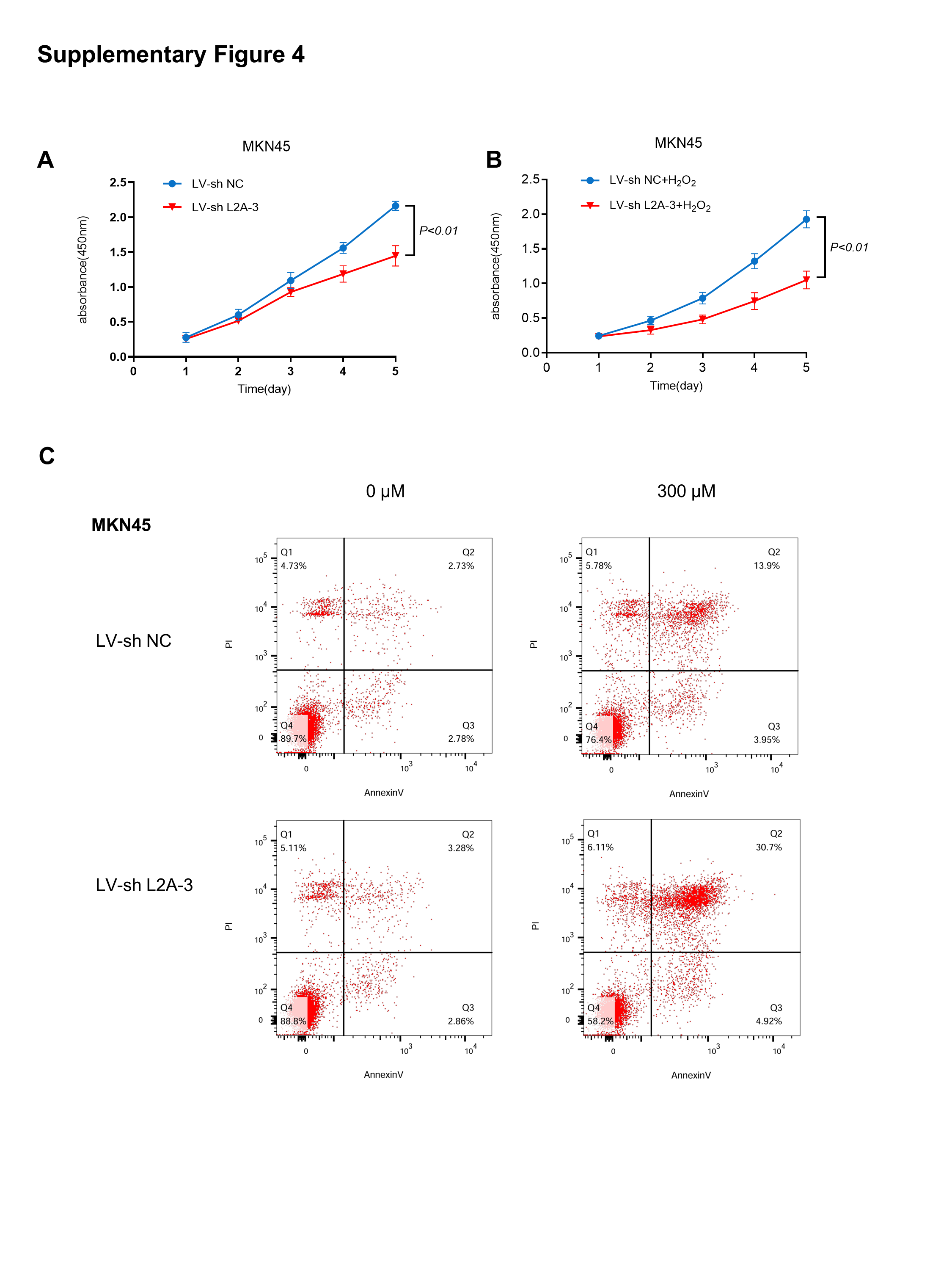

Supplement: S4 Fig — (A) CCK-8 proliferation assay of LV‑shL2A‑3 and LV‑shNC cells. (B) Proliferation of LV‑shL2A‑3 and LV‑shNC cells after treatment with 150 μM H₂O₂. (C) Flow cytometry analysis of apoptosis in LV‑shL2A‑3 and LV‑shNC cells following exposure to H₂O₂ (0, 300 μM) for 24 hours. Consistent with the results obtained with LV‑shL2A‑2, cells transfected with LV‑shL2A‑3 exhibited significantly increased sensitivity to H₂O₂-induced oxidative stress. Data are presented as mean±SD of three independent experiments. ns, no significance, *P < 0.05. **P < 0.01. (TIF) [file pone.0331823.s004.tif]
