# Supplementary material for: LAMP2A-dependent chaperone-mediated autophagy enhances oxidative stress resistance in gastric cancer cells through selective degradation of accumulated oxidized DJ-1
Source: PLoS One. 2026 May 15;21(5):e0331823. doi: 10.1371/journal.pone.0331823 (PMC13178873; doi:10.1371/journal.pone.0331823)
Supplement: Attachment — Submitted filename: Response to Reviewers.docx [file pone.0331823.s006.docx]

Dear Editors,

On behalf of all the contributing authors, I appreciate the time and effort that you and the reviewers dedicated to providing feedback on our manuscript. In addition, I am grateful for the insightful comments concerning our manuscript entitled “LAMP2A-dependent chaperone-mediated autophagy enhances oxidative stress resistance in gastric cancer cells through selective degradation of accumulated oxidized DJ-1” (Manuscript ID PONE-D-25-43608). As a young researcher, I felt fully prepared when submitting the manuscript, but the revision feedback each time reveals there's still so much I need to improve. I sincerely thank the editors and reviewers for their guidance - I can truly feel myself growing through this process.

According to the editors’ and reviewers’ comments, we have made extensive modifications to our manuscript. In this revised version, all changes to our manuscript are highlighted in red within the document. Our point-by-point responses to the reviewers are listed below this letter.

Once again, we thank the reviewers and editors for their insightful comments. We hope this revised manuscript can meet your requirements, and we are looking forward to your reply.

Sincerely,

Shuangshuang Le

**Reviewer #1:** The manuscript examines the role of chaperone-mediated autophagy (CMA) in gastric cancer under conditions of oxidative stress. The authors demonstrate that LAMP2A, the rate-limiting component of CMA, is upregulated in gastric cancer tissues and further induced upon exposure to H₂O₂. Knockdown of LAMP2A sensitises gastric cancer cells to apoptosis triggered by oxidative stress, whereas its overexpression promotes cell survival. They propose that CMA selectively degrades hyperoxidised DJ-1, thereby maintaining redox homeostasis and protecting tumour cells. The study concludes that the interaction between LAMP2A and DJ-1 represents a key mechanism conferring resistance to oxidative stress in gastric cancer cells.

**Major comments:**

**Comment 1**

The central claim that hyperoxidised DJ-1 is a CMA substrate is not fully substantiated; the oxidation status of DJ-1 was inferred indirectly and not directly measured (for example, using specific antibodies, redox-sensitive mutants, or mass spectrometry).

**Response:** We sincerely thank the reviewer for this insightful and critical comment regarding the central claim of our study. We fully acknowledge that direct measurement of DJ-1 hyperoxidation would provide the most definitive validation.

**The over-oxidized form of DJ-1:** DJ-1 is a protein that protects cells from oxidative stress. Its function depends on a specific amino acid, Cysteine 106 (Cys-106).

**Normal State:** Under normal conditions, Cys-106 is in a reduced state (Cys-SH), which is essential for DJ-1's protective role.

**Mild Oxidation:** When cells experience mild oxidative stress, Cys-106 can be reversibly oxidized (e.g., to Cys-SOH). This can act as a sensor and signal.

**Over-Oxidation (Irreversible Oxidation):** Under intense or chronic oxidative stress, Cys-106 undergoes irreversible over-oxidation to Cys-sulfinic acid (Cys-SO₂H). This form loses the protein's protective function and is often associated with diseases like Parkinson's.

**Figure 1** Oxidation of cysteine residue in DJ 1. Cys 106 of DJ1 is sequen tially oxidized to SOH, SO2H, and SO3H. [1]

**Methods for Detecting DJ-1 Oxidation Forms**

**The most Common Method for detecting the over-oxidized form of DJ-1 is 2D Gel Electrophoresis.** This is the best gel-based method. It separates proteins by charge first, then by size. Over-oxidized DJ-1 appears as a more acidic spot to the left of the normal DJ-1 spot.

**Figure 2** The oxidation of DJ-1 was analyzed using isoelectric focusing electrophoresis followed by western blotting with an anti-DJ-1 antibody [2].

**The most Precise Method for detecting the over-oxidized form of DJ-1 is Mass Spectrometry.** This method provides definitive proof by measuring the exact mass change, which can confirm the exact site of modification.

While such techniques (e.g., 2D Gel Electrophoresis or mass spectrometry) were beyond the scope of our current technological landscape, we have built a compelling, albeit indirect, case based on multiple convergent lines of evidence.

**Our conclusion is based on the following logical reasoning:**

It is well-established that severe oxidative stress (300 µM H₂O₂) converts DJ-1 from a functional form to a dysfunctional, hyperoxidized state (SO₃H) [32, 33].

Under this same stress, we observed enhanced binding between LAMP2A and DJ-1 (Fig. 5D, E). Knocking down LAMP2A under these conditions caused DJ-1 accumulation and activated apoptosis (Fig. 6).

The most coherent explanation is that CMA is selectively degrading the toxic, hyperoxidized DJ-1. This resolves the paradox of why a protective protein would be degraded under stress—CMA is removing its damaged, harmful form.

We have added the following statement to the Discussion to explicitly note this limitation: **"****We acknowledge that the designation of hyperoxidized DJ-1 as a CMA substrate is based on indirect evidence. Directly identifying the targeted oxidized species represents an essential goal for future work, potentially through techniques like redox-specific 2D gel electrophoresis or mass spectrometry."**

We appreciate this insightful comment, which has helped us improve the clarity and rigor of our manuscript.

**Comment 2**

Rescue experiments are lacking. Demonstrating that shRNA-resistant LAMP2A can reverse the observed phenotypes would considerably strengthen the conclusions.

**Response:** This is an excellent suggestion. We have successfully performed this key rescue experiment. We constructed a shRNA-resistant LAMP2A plasmid and co-transfected it into our LAMP2A-knockdown MKN45 cells. The new results (now included as Figure 6B) demonstrate that re-expression of LAMP2A significantly reversed the increased DJ-1 and the dysregulation of BAX/Bcl-2 caused by LAMP2A knockdown under oxidative stress（H_2_O_2_：300uM）. This strongly confirms the specific role of LAMP2A in mediating the observed phenotypes.

**Figure 6B**

**Comment 3**

Controls using antioxidants (e.g., NAC, GSH donors) are not included, making it unclear whether the observed effects are truly ROS-dependent.

**Response:** We sincerely thank the reviewer for this insightful comment. We agree that the inclusion of antioxidant controls, such as N-acetylcysteine (NAC), would further strengthen the evidence for ROS-dependency. Our new data (now included as Supplementary Figure 1) demonstrate that NAC pretreatment effectively prevented the H₂O₂-induced upregulation of both LAMP2A and DJ-1. This provides direct evidence that the observed effects are specifically mediated by ROS.

**Figure S1**

**Comment 4**

The authors equate LAMP2A knockdown with CMA inhibition, but more direct assessment of CMA activity is necessary (for example, KFERQ reporter assays or lysosomal uptake assays).

**Response:** We agree with the reviewer that a direct CMA flux assay would be valuable. However, establishing the KFERQ reporter assay or similar sophisticated lysosomal uptake assays in our lab has proven to be technically challenging and time-consuming within the revision period. Direct and consistent evidence from seminal studies shows that genetic ablation of LAMP2A blocks CMA flux, establishing it as a definitive and specific method to inhibit this pathway.

For example,

1. “Lysosome-associated membrane protein 2A (LAMP2A) was knocked down or overexpressed to assess the effects of hepatocyte-specific CMA on NASH progression.” [1]

2. “Silencing of LAMP2A to inhibit CMA activity reversed 922-induced IGF-1Rβ degradation, suggesting that IGF-1Rβ degradation by 922 was partially dependent on the CMA pathway rather than macroautophagy” [2].

3. “Lysosome-associated membrane protein 2a (Lamp2a) overexpression and knockdown were used to causally study CMA's role in hypoxically stressed cardiomyocytes. LAMP2a protein levels were used as both a primary indicator and driver of CMA function [3].”

4. “CMA activity was increased in SW480 and HT29 colorectal cancer cells with a LAMP2A overexpression vector and CMA activity was decreased using a LAMP2A short interfering RNA vector [4].”

To address the reviewer's concern and improve the clarity of our manuscript, we provided a more nuanced explanation in the Results section (3.4). The revised text now reads:**"LAMP2A knockdown significantly impaired cellular proliferative capacity and augmented apoptosis under oxidative stress. Since LAMP2A is the limiting receptor for CMA, its knockdown is expected to, and has been widely demonstrated to, inhibit CMA activity [15, 19]. "**

We hope that this clarification, backed by established literature and our own functional data, adequately addresses the reviewer's concern.

**Minor comments:**

**Comment 1**

The manuscript would benefit from language editing to reduce repetition and improve clarity.

**Response:** We thank the reviewer for this helpful comment. Acknowledging the need for improved clarity, we have had the manuscript reviewed by a native English-speaking colleague in our field. They have corrected grammatical errors and enhanced the overall fluency, with all changes highlighted using "Track Changes" for the reviewer's convenience.

**Comment 2**

Figure legends lack methodological details such as number of replicates and statistical tests. Quantification of western blot bands should also be included.

**Response:** We have updated all figure legends to explicitly state the number of independent biological replicates (e.g., n=3) and the specific statistical tests used (e.g., Student’s t-test, one-way ANOVA). Quantitative data from Western blots (densitometry analysis) are now presented as bar graphs beneath the representative blot images in all relevant figures.

**Comment 3**

Justification for selecting 150 µM and 300 µM H₂O₂ concentrations is required, as toxicity may vary across cell lines.

**Response:** We have added a justification in the Methods section (*2.6 H₂O₂-Induced Oxidative Stress Model Construction*). The concentrations were selected based on preliminary CCK-8 assays to identify doses that induce sub-lethal (150 µM) and severe (300 µM) oxidative stress across the four cell lines, resulting in a measurable but not overwhelming reduction in cell viability (approximately 20-30% and 40-60% reduction after 24h, respectively), allowing us to observe both adaptive and cytotoxic responses.

**Comment 4**

Negative controls in co-IP and immunofluorescence experiments should be clearly described (for example, IgG controls, secondary-only staining).

**Response:** We sincerely thank the reviewer for this valuable comment. We have now provided detailed descriptions of the negative controls used in both the co-immunoprecipitation (Co-IP) and immunofluorescence (IF) experiments in the revised manuscript.

The specific modifications made to the manuscript are as follows:

1. In the Methods section (2.10 Co‑immunoprecipitation analysis), we have added:

**“As a negative control, normal rabbit IgG (1:1000, 2729, Cell Signaling Technology) was used in place of the primary antibody under identical conditions to confirm the specificity of the antibody-protein interaction.”**

2. In the Methods section (2.8 Immunofluorescence), we have added:

**“****To control for non-specific binding of the secondary antibody, a negative control omitting the primary antibody was included in each experiment.”**

**Comment 5**

Some references are outdated; inclusion of recent studies on CMA and DJ-1 in cancer would strengthen the background.

**Response:** We sincerely thank the reviewer for this valuable comment. We have updated the reference list, adding several key recent publications on CMA in cancer (PMID: 35435804, PMID: 35267139, PMID: 37509689, PMID: 38104013) and the role of DJ-1 (PMID: 40327696, PMID: 12446870, PMID: 37480966, PMID: 36678610, PMID: 39155875, PMID: 27171370) in the Introduction and Discussion.

**Comment 6**

The data availability statement (“not publicly available due to privacy of laboratory content”) is inconsistent with PLOS ONE’s open data policy and must be corrected.

**Response:** We apologize for this oversight. The data availability statement has been revised to fully comply with PLOS ONE's policy: **"****All data generated or analysed during this study are included in this published article and its supplementary information files. The raw datasets are also available from the corresponding author on reasonable request."**

**Reviewer #2: The manuscript by Shuangshuang Le and co-workers describes the role of LAMP2A in gastric cancer under oxidative stress condition. The topic is interesting; however, some points should be clarified.**

**Comment 1**

The overexpression of LAMP2A in gastric cancer has been demonstrated in the reference 14 (Zhou J et al., 2016). Authors should clarify the novelty of their results shown in Figure 1 compared to the previous published data.

**Response:** We thank the reviewer for pointing this out. While Zhou et al. established LAMP2A upregulation in GC, our study builds upon this by specifically investigating its dynamic regulation under oxidative stress and its functional consequences for cell survival, which was not the focus of the previous work. Our data confirms and extends previous findings by linking LAMP2A expression to the oxidative stress response.

To clarify this point for readers, we have added the following sentence to the results section of the revised manuscript (Section3.1 Upregulation of LAMP2A in Gastric Cancer Promotes CMA):

" **Consistent with previous reports [14], we confirmed that LAMP2A was upregulated in gastric adenocarcinoma tissues and cell lines (Fig. 1A-D). Given that LAMP2A is the rate-limiting factor of CMA, its elevated level indicates enhanced CMA activity in gastric cancer cells compared to normal gastric mucosa cells.** "

We hope this clarification satisfactorily addresses the reviewer's concern regarding the novelty of our work.

**Comment 2**

Authors declare that LAMP2A knockdown inhibit CMA activity in their experimental model. To support this conclusion, authors should analyse additional CMA markers such as the expression of Hsc70 on lysosomes.

**Response:** We thank the reviewer for this insightful comment. As mentioned in our response to Reviewer #1 (Comment 4), we agree that multiple lines of evidence are ideal for confirming CMA activity. While we did not directly measure lysosomal HSC70 in this study, we based our conclusion on two strong and established pieces of evidence:

1. **Well-Established Role of LAMP2A:** It is widely accepted in the CMA field that the abundance of LAMP2A at the lysosomal membrane is the rate-limiting step for CMA activity. Direct and consistent evidence from seminal studies shows that genetic ablation of LAMP2A blocks CMA flux, establishing it as a definitive and specific method to inhibit this pathway.

For example,

a. “Lysosome-associated membrane protein 2A (LAMP2A) was knocked down or overexpressed to assess the effects of hepatocyte-specific CMA on NASH progression.” [1]

b. “Silencing of LAMP2A to inhibit CMA activity reversed 922-induced IGF-1Rβ degradation, suggesting that IGF-1Rβ degradation by 922 was partially dependent on the CMA pathway rather than macroautophagy” [2].

c. “Lysosome-associated membrane protein 2a (Lamp2a) overexpression and knockdown were used to causally study CMA's role in hypoxically stressed cardiomyocytes. LAMP2a protein levels were used as both a primary indicator and driver of CMA function [3].”

d. “CMA activity was increased in SW480 and HT29 colorectal cancer cells with a LAMP2A overexpression vector and CMA activity was decreased using a LAMP2A short interfering RNA vector [4].”

2. **Functional Evidence from CMA Substrate Accumulation:** A key functional indicator of impaired CMA is the accumulation of known CMA substrates. In our study, we observed a significant increase in the total protein level of DJ-1 upon LAMP2A knockdown (Fig. 6). This accumulation is consistent with DJ-1 being a CMA substrate and its degradation being blocked when CMA is inhibited, thus serving as indirect but compelling functional evidence for reduced CMA activity in our model.

**Comment 3**

Authors should better describe the results of Figure 4C and D. Dot plots show that in both downregulated and overexpressed LAMP conditions, the number of double-positive cells increase compared to the control suggesting that both conditions enhance the percentage of dying cells under oxidative stress. How can these data be explained?

**Response:** We sincerely thank the reviewer for this astute observation and the opportunity to clarify our data interpretation. The reviewer is correct in noting an increase in double-positive cells in certain conditions, and we agree that this requires a clear explanation based on the principles of Annexin V/7-AAD staining.

With this in mind, we re-analyzed our data:

1. In LAMP2A-knockdown cells (Fig. 4E): As the reviewer noted, there is an increase in double-positive cells. However, the most dramatic and statistically significant change is the substantial increase in the population of early apoptotic cells (Annexin V+/7-AAD-) upon H₂O₂ treatment. This indicates that LAMP2A knockdown potently initiates the apoptotic process. The concomitant increase in late apoptotic cells is a logical consequence, as these early apoptotic cells rapidly progress to a late stage due to the severe oxidative insult. The total apoptosis rate (sum of early and late apoptotic cells) is significantly higher in the knockdown group, which is the standard metric for analysis.
2. In LAMP2A-overexpression cells (Fig. 4F): LAMP2A overexpression potently inhibits the initiation of apoptosis, as unequivocally shown by the significant reduction of cells in the early apoptotic (Annexin V+/7-AAD-) quadrant in Fig. 4F. However, cells that ultimately cannot be rescued under such severe stress might undergo a very rapid transition from a healthy state to late-stage death, bypassing a detectable early apoptotic phase. Consequently, at the 24-hour time point measured, a higher proportion of the dying population in the overexpressing group is captured in the late apoptotic/necrotic stage.

To prevent any potential misunderstanding, we have revised the relevant results section (3.4) in the manuscript to provide a more detailed description: **"****Flow cytometric analysis revealed that LAMP2A-knockdown significantly increased the proportion of cells in early apoptosis (Annexin V+/7-AAD-) upon H₂O₂ exposure, which was accompanied by a subsequent rise in late apoptotic/necrotic cells (Annexin V+/7-AAD+), leading to a dramatic increase in the total apoptosis rate (Fig. 4E). Conversely, LAMP2A-overexpression notably reduced the early apoptotic population under oxidative stress, demonstrating a protective effect against apoptosis initiation. Its total apoptosis rate was still significantly lower than the control (Fig. 4F)."**

**Reviewer #3: DJ-1 (PARK7) is known to protect cells against oxidative stress and is a target for degradation by chaperone-mediated autophagy (CMA). In the present manuscript, the authors examined the involvement of DJ-1 (PARK7) in LAMP2A-mediated oxidative stress resistance in gastric cancer cells. The authors demonstrated that both LAMP2A and DJ-1 were upregulated in gastric cancer cells and H2O2-induced oxidative stress further increased their levels. LAMP2A and DJ-1 interacted with each other, and DJ-1 contains a conserved KFERQ-like motif, suggesting DJ-1 is a substrate for CMA. However, inhibition of CMA by LAMP2A knockdown caused an increase in DJ-1 which was associated with a decrease in the antiapoptotic protein Bcl2 and an increase in the pro-apoptotic protein BAX. The authors proposed that oxidative-stressed induced clearance of hyperoxidized DJ-1 is responsible for gastric cancer cell survival.**

The results are straightforward but requires additional study to substantiate their conclusion.

**Comment 1**

While the results show that an increase in DJ-1 by LAMP2A alters the ratio of pro- and anti-apoptotic proteins, no evidence has been provided to support that there are two forms of DJ-1 and the hyperoxidized form of DJ-1 promotes apoptosis. Does the extent of LAMP2A knockdown affects the outcome?

**Response:** We thank the reviewer for raising this important point regarding the direct demonstration of distinct DJ-1 isoforms. Reviewer 1 also raised a similar point.We acknowledge that providing a direct detection of the hyperoxidized DJ-1 form in our system would further strengthen the manuscript. While such experiments (e.g., using isoform-specific antibodies or 2D gels) are technically challenging and beyond the scope of the current revision, we have built a compelling, albeit indirect, chain of evidence to support our conclusion:

Our model is supported by three key evidences: First, the enhanced LAMP2A-DJ-1 interaction under oxidative stress (Fig. 5D, E) indicates that modified DJ-1 is recognized by CMA. Second, the concurrent DJ-1 accumulation and increased apoptosis upon LAMP2A knockdown (Fig. 6A) is paradoxical if DJ-1 is protective, implying the accumulated pool contains a pro-apoptotic, hyperoxidized form. This is consistent with established literature showing hyperoxidized DJ-1 loses its anti-apoptotic function [32]. While directly identifying the DJ-1 isoform remains a future goal, this evidence solidly supports our model.

To address whether the extent of LAMP2A knockdown affects the outcome, Quantitative analysis of our Western blot data revealed a clear dose-dependency: more efficient LAMP2A knockdown correlated with greater DJ-1 accumulation and a higher Bax/Bcl-2 ratio. This confirms that the phenotypic severity is directly linked to the degree of CMA impairment.

**Comment 2**

At least 2 different LAMP2A shRNA should be used in all experiments.

**Response:** We sincerely thank the reviewer for raising this critical point regarding the specificity of our knockdown experiments. We completely agree that using multiple distinct shRNAs is essential to rule out off-target effects and to robustly link the observed phenotypes to the specific knockdown of LAMP2A.

To rule out off-target effects, we validated our knockdown phenotypes using a second, independent shRNA (shLAMP2A#2). Remarkably, all functional assays repeated with this second shRNA produced results consistent with those obtained with the original shRNA, thereby substantiating the specificity of LAMP2A loss-of-function (Supplementary Figure 2A, B, C).

**Figure S2**

**Comment 3**

In Fig. 1, the protein level of MKN28 is more than AGS but in Fig. 2, H2O2 had no effect on LAMP2A expression in MKN28 cells but caused a substantial increase in AGS cells. In Fig 1C and 1D, NKM28 should be MKN28.

**Response:** We thank the reviewer for their careful observation. The reviewer astutely observes that while MKN28 cells have a higher basal level of LAMP2A than AGS cells under normal conditions (Fig. 1D), they show a blunted response to H₂O₂-induced upregulation compared to AGS cells (Fig. 2). We believe it is not a contradiction but rather a reflection of cell line-specific biological contexts. Different gastric cancer cell lines harbor distinct genetic backgrounds and signaling pathway activations. AGS cells, with moderate basal LAMP2A, may possess a more responsive signaling cascade (e.g., involving Nrf2, HIF1α, or other stress-responsive transcription factors) that allows for robust induction upon H₂O₂ challenge.

We also apologize for the typo; "NKM28" has been corrected to "MKN28" in Figures 1C and 1D.

**Comment 4**

Fig. 4D: LAMP2A KD increased apoptosis and decreased cell proliferation in MKN45 cells in response to H2O2. While LAMP2A overexpression in AGS cells attenuated early apoptotic cells following H2O2 treatment, the late apoptotic and/or necrotic cells were increased with little change in the healthy cells (B3). What is the effect of LAMP2A overexpression on cell proliferation?

**Response:** We thank the reviewer for this insightful question regarding the functional outcome of LAMP2A overexpression on cell proliferation. In response, we have now included the CCK-8 proliferation assay data for the LAMP2A-overexpressing AGS cells in the revised manuscript as new panel C, D in Figure 4. Under treatment with 150 µM H₂O₂, AGS cells overexpressing LAMP2A exhibited a markedly higher proliferation rate compared to the negative control (NC) cells. The increase in late apoptotic/necrotic cells is likely a phenomenon observed under the specific, severe condition of 150/300 µM H₂O₂ at the 24-hour endpoint. It may represent a minor population of cells that, despite the CMA-mediated protection, ultimately succumb to stress via alternative death pathways. However, the key finding is that the majority of the population is rescued from early apoptotic commitment, the CCK-8 proliferation assay data for the LAMP2A-overexpressing AGS cells provides clear functional evidence.

**Figure4**

**Comment 5**

Fig. 5A: DJ-1 is increased to a similar extent in AGS, MKN45 and MKN28 cells.

**Response:** We thank the reviewer for this insightful observation. This finding indicates that DJ-1 is universally upregulated in gastric cancer cells. However, as shown in our Results 3.1 and 3.2, the expression level of LAMP2A varies among different cell lines, indicating that the expression patterns of DJ-1 and LAMP2A are not entirely consistent across gastric cancer cell lines. We argue that the universal upregulation of DJ-1 does not contradict the cell type-specific expression of LAMP2A; instead, it precisely underscores the core of our proposed mechanism: DJ-1 accumulation represents a common consequence of oxidative stress, whereas LAMP2A-mediated CMA serves as a specific protective mechanism that clears this damage and determines cell survival. The differential ability of various cell lines to activate LAMP2A is key to their distinct stress resistance capabilities.

**Comment 6**

Fig. 6: What is the effect of LAMP2A overexpression on DJ-1 level and apoptosis?

**Response:** We thank the reviewer for this important question. In direct response to your comment, we performed additional experiments to assess the effect of LAMP2A overexpression on DJ-1 and apoptosis-related proteins. The new data, now included in the revised manuscript as Figure 6B. The new results (now included as Figure 6B) demonstrate that re-expression of LAMP2A significantly reversed the increased DJ-1 and the dysregulation of BAX/Bcl-2 caused by LAMP2A knockdown under oxidative stress (H2O2：300uM).

**Comment 7**

Discussion, Pg. 15, 2nd para: The sentence “Tumor microenvironment….CMA activity” should be corrected.

**Response:** We thank the reviewer for this correction. The original sentence has been revised. The corrected sentence now reads: **"****Furthermore, stressors such as nutrient deprivation, hypoxia, and ROS have been demonstrated to serve as potent inducers of CMA activity [22]."**

**Comment 8**

Pg. 16, 2nd para: DJ-1 is not a new antioxidant molecule, and it is also not a recently discovered molecule. In fact, Ref. 33 published in 2004 is focused on DJ-1. Is the antioxidant function of DJ-1 recently discovered?

**Response:** We sincerely thank the reviewer for this correction. The reviewer is absolutely right. We apologize for the imprecise language in our original manuscript. DJ-1 (PARK7) is indeed a well-established protein, and its canonical cytoprotective and antioxidant functions have been extensively studied for decades, as correctly referenced by the reviewer.

To address this, we have thoroughly revised the relevant paragraph on Page 16 to eliminate any misleading claims. The text now clearly states: **“DJ-1 was first discovered in 1997 as an oncogene and was associated with early-onset PD in 2003 [24]. Since then, DJ-1, as an antioxidant stress protein, has attracted increasing attention. DJ-1 can eliminate intracellular ROS through self-oxidation. DJ-1 can activate intracellular multiple molecular signaling pathways and enhance the expression of cellular antioxidant enzymes and promote the elimination of ROS [25].”**

We thank the reviewer again for prompting this important clarification, which has significantly improved the accuracy of our discussion.

**Comment 9**

Pg. 17, 1st para: The sentence “Given DJ-1's established cytoprotective properties through redox homeostasis maintenance and apoptosis inhibition” is incomplete.

**Response:** We thank the reviewer for catching this grammatical error. The sentence was indeed incomplete and has been corrected in the revised manuscript. The revised text now reads as a complete sentence, logically connecting the established role of DJ-1 with the central paradox of our study: **“Although DJ-1 is well-known for protecting cells by maintaining redox balance and inhibiting apoptosis, we found that CMA, another cytoprotective pathway, targets DJ-1 for degradation. This presents an apparent paradox.”**

**Comment 10**

Pg. 18: It was mentioned that “CMA may regulate neuronal mitochondrial homeostasis by degrading oxidatively damaged DJ-1 protein, as demonstrated in a recent study [34].” Is an alteration in mitochondrial homeostasis caused by the degradation of oxidatively damaged DJ-1 responsible for its effects on pro- and anti-apoptotic proteins? Ref 34 was published almost 10 years ago and not recently.

**Response:** We sincerely thank the reviewer for these critical corrections. The reviewer is absolutely correct. The use of the word "recent" to describe the 2016 study by Wang et al. was inaccurate, especially in the fast-moving field of biomedical research. We have removed the word "recent" from the sentence in the revised manuscript.

Regarding the mechanism, our study did not directly test if mitochondrial dysfunction is the required intermediate step. Instead, our data demonstrate that the accumulation of hyperoxidized DJ-1 itself, resulting from impaired CMA, is sufficient to trigger the pro-apoptotic shift in BAX and BCL-2 in our gastric cancer model. While the mitochondrial pathway is a plausible downstream effect, our findings highlight a more direct causal link between DJ-1 accumulation and apoptosis initiation.

**Reviewer #4: In the current manuscript the authors have examined the role of DJ-1 and LMAP2 mediated autophagy in gastric cancer cells. Authors have reported the significance of DJ-1 and LAMP2 interaction in promotion of gastric carcinoma. The overall manuscript is well written. However, authors need to address the following concerns:**

**Comment 1**

LAMP2-DJ1 interaction is not convincing. The arrangement of IP blot is confusing. The IF image is of very poor quality. More over LAMP2 protein is localized in the lysosomal or late endosomal membrane not in the nucleus where most of the DJ1 is localized after H2O2 treatment.

**Response:** We sincerely thank the reviewer for these critical and insightful comments.

1. We have re-organized the Co-IP blot in Figure 5D for better clarity, clearly marking Input, IgG control, and IP lanes.
2. We have replaced the IF image in Figure 5E with a higher-resolution, clearer confocal image.
3. We thank the reviewer for this critical point. We agree that LAMP2A, as a lysosomal membrane protein, is not nuclear. The co-localization is perinuclear, not nuclear. We think that the images show that LAMP2A and DJ-1 co-localize in the perinuclear region. Studies indicate that DJ-1 translocates to the nucleus under oxidative stress to execute cytoprotective functions [5]. The distribution of lysosomes, central hubs for cellular energy sensing and regulation, is precisely controlled within cells, primarily exhibiting two distinct localization patterns: perinuclear and peripheral [6]. Our confocal imaging data under oxidative stress conditions reveal that CMA recognizes and degrades DJ-1 at the surface of perinuclear lysosomes.

**Figure 5E**

**Comment 2**

To further confirm the LAMp2 and DJ1 localization authors need to perform PLA or other similar kind of assay.

**Response:** We thank the reviewer for this valuable suggestion to further strengthen our localization data using a proximity ligation assay (PLA). We agree that this technique would provide superior spatial resolution to confirm the LAMP2A-DJ-1 interaction.

However, establishing the Proximity Ligation Assay (PLA) in our laboratory reliably within the revision timeframe has proven to be a significant technical challenge. We have explicitly acknowledged this limitation and the reviewer's insightful suggestion in the revised discussion section.

We hope that the improved Co-IP and immunofluorescence data, which now more clearly demonstrate the interaction and colocalization, will be sufficient to support our conclusions at this stage.

**Comment 3**

Since the protein expression level varies among different cell types, it is not ideal to compare the only LAMP2 expression in Fig 1D. To establish the higher expression, authors need to include other autophagy related proteins too.

**Response:** We thank the reviewer for this valuable comment. We agree that comparing absolute protein levels across different cell lines requires caution. Our conclusion of enhanced CMA in gastric cancer is not based solely on Fig. 1D, but is supported by multiple lines of evidence:

1. Elevated LAMP2A in clinical GC tissues (IHC, Fig. 1A).
2. Upregulated LAMP2A mRNA in GC cohorts (TCGA/GEO, Fig. 1B).

Critically, LAMP2A is the established rate-limiting factor for CMA activity (refs #15, #23). Therefore, its consistent and generally higher expression across gastric cancer cell lines provides meaningful support for enhanced CMA potential in gastric cancer.

To address the reviewer's point, we have revised the text to focus on the consistent presence of LAMP2A, rather than direct quantitative comparisons between cell lines.

**Comment 4**

H2O2 at higher dose can activate various signaling pathways, therefore authors need to repeat the main experiments in presence of another inducer and perform the rescue experiment with NAC.

**Response:** We thank the reviewer for this important suggestion. In response, we have performed key rescue experiments using the ROS scavenger NAC.

Our new data (now included as Supplementary Figure 2) demonstrate that NAC pretreatment effectively prevented the H₂O₂-induced upregulation of both LAMP2A and DJ-1. This provides direct evidence that the observed effects are specifically mediated by ROS.

**Figure S1**

Regarding the use of an alternative oxidative stress inducer, we agree it would be valuable. While repeating all experiments with another inducer falls beyond the current scope of this study, we have acknowledged this point in the revised Discussion as a direction for future research.

We believe the NAC rescue experiment robustly addresses the core of the reviewer's concern by establishing a causal role for ROS.

**Comment 5**

After LAMP2 over expression, the DJ1 protein level needs to be examined.

**Response:** We thank the reviewer for this suggestion. We have now examined DJ-1 levels following LAMP2A overexpression. This new finding provides compelling and symmetrical evidence for our conclusion: Knockdown of LAMP2A impairs DJ-1 degradation, leading to its accumulation (as shown in Fig. 6A). Overexpression of LAMP2A enhances DJ-1 degradation, leading to its reduction (as shown in Fig. 6B).

**Figure 6B**

**Comment 6**

The Fig 1A, the histology of the cancer samples are completely different than the control. the authors need to provide more information about the patient sample collection procedure.

**Response:** We thank the reviewer for this comment. The histological difference between cancer and normal samples in Fig 1A is expected, as the commercial tissue microarray (Shanghai Outdo Biotechnology, HStmA020PG01) contains paired primary gastric cancers and adjacent non-tumor tissues from the same patients. The adjacent tissues were pathologically confirmed to be cancer-free. We have added these details to the Methods section (*2.1Tissue microarray analysis*) to provide full transparency about the sample source.

**Comment 7**

There is no text in under section 3.7.

**Response:** We apologize for this oversight. Section 3.7, "Mechanisms of LAMP2A-dependent CMA enhances oxidative stress resistance in gastric cancer cells," was intended as a brief summary leading into the Discussion (Page 13). We have now moved its content to the beginning of the Discussion to improve the manuscript's flow and removed the empty section 3.7.

**Comment 8**

150 uM of H2O2 is quite high and after 5 days of treatment there is not much significant difference in cell proliferation in figure 4A &B which is confusing.

**Response:** We thank the reviewer for this insightful observation. The experimental design for Figure 4B may have been unclear. In our experimental design to investigate the effects of prolonged oxidative stress on tumor cells, the treatment duration was extended to 5 days. Due to the inherent instability and rapid decomposition of H₂O₂, and to ensure sustained cell viability under 150 μM H₂O₂ stimulation, we adopted an intermittent exposure protocol.

Specifically, to model chronic, intermittent oxidative stress (which better reflects the fluctuating tumor microenvironment than continuous exposure), cells were treated with 150 µM H₂O₂ for 8 hours per day, after which the medium was replaced with fresh, H₂O₂-free medium. The key finding is that under this recurring stress, LAMP2A-knockdown cells exhibit a significantly impaired ability to adapt and proliferate compared to controls.

We acknowledge that the differences are moderate, as both cell types can partially recover. This precisely demonstrates that CMA is critical for maintaining fitness under cyclic stress, not just for surviving acute insult. We will clarify this experimental design and interpretation in the revised manuscript. The more dramatic effect of LAMP2A knockdown on acute sensitivity is robustly demonstrated in the apoptosis assay (Fig 4C).

**References**

1. Yoshiro Saito. Oxidized DJ-1 as a possible biomarker of Parkinson's disease. Journal of Clinical Biochemistry and Nutrition. 54(3):138-144.
2. Ji Cao, Meidan Ying, Nan Xie, Guanyu Lin, Rong Dong, Jun Zhang, Hailin Yan, Xiaochun Yang, Qiaojun He, Bo Yang. The oxidation states of DJ-1 dictate the cell fate in response to oxidative stress triggered by 4-hpr: autophagy or apoptosis? Antioxid Redox Signal 21(10):1443-1459 (2014).
3. Shuoyi Ma, Erzhuo Xia, Miao Zhang, Yinan Hu, Siyuan Tian, Xiaohong Zheng, Bo Li, Gang Ma, Rui Su, Keshuai Sun, Qingling Fan, Fangfang Yang, Guanya Guo, Changcun Guo, Yulong Shang, Xinmin Zhou, Xia Zhou, Jingbo Wang, Ying Han. Role of the FOXM1/CMA/ER stress axis in regulating the progression of nonalcoholic steatohepatitis. Clin Transl Med 15(2): e70202 (2025).
4. Nina Xue, Fangfang Lai, Tingting Du, Ming Ji, Di Liu, Chunhong Yan, Sen Zhang, Xiaoming Yu, Jing Jin, Xiaoguang Chen. Chaperone-mediated autophagy degradation of IGF-1Rβ induced by NVP-AUY922 in pancreatic cancer. Cell Mol Life Sci 76(17): 3433-3447 (2019).
5. Rajeshwary Ghosh, Jennifer Jason Gillaspie, Kenneth S Campbell, J David Symons, Sihem Boudina, James Scott Pattison. Chaperone-mediated autophagy protects cardiomyocytes against hypoxic-cell death. Am J Physiol Cell Physiol 323(5): C1555-C1575 (2022).
6. Rui Chen, Yanfei Zhang, Yuanxun Ge, Chao He, Zongyao Wu, Junhua Wang, Jin Yu, Jing Xiao, Xu Zhang, Minghua Tao, Zi Wang, Li Pan, Meng He, Shuhui Li, Qi Han. LAMP2A overexpression in colorectal cancer promotes cell growth and glycolysis via chaperone‑mediated autophagy Oncol Lett 27(1):33 (2023).
7. Asier González 1, Michael N Hall 1, Sheng-Cai Lin 2, D Grahame Hardie. AMPK and TOR: The Yin and Yang of Cellular Nutrient Sensing and Growth Control. Cell Metabolism 3;31(3):472-492 (2020).
8. Zhiquan Wang, Jun Liu, Siyan Chen, Ying Wang, Li Cao, Yu Zhang, Wenyan Kang, Hui Li, Yaxing Gui, Shengdi Chen, Jianqing Ding. DJ-1 modulates the expression of Cu/Zn-superoxide dismutase-1 through the Erk1/2-Elk1 pathway in neuroprotection. Annals of Medicine 70(4):591-599 (2011)
