# Supplementary material for: LAMP2A-dependent chaperone-mediated autophagy enhances oxidative stress resistance in gastric cancer cells through selective degradation of accumulated oxidized DJ-1
Source: PLoS One. 2026 May 15;21(5):e0331823. doi: 10.1371/journal.pone.0331823 (PMC13178873; doi:10.1371/journal.pone.0331823)
Supplement: Attachment — Submitted filename: Response to Reviewers .docx [file pone.0331823.s007.docx]

Dear Editors,

On behalf of all authors, we sincerely thank you for the opportunity to revise our manuscript titled “LAMP2A-dependent chaperone-mediated autophagy enhances oxidative stress resistance in gastric cancer cells through selective degradation of accumulated oxidized DJ-1” (ID: PONE-D-25-43608). We are grateful for the constructive and insightful comments from the reviewers, which have helped us significantly improve the clarity, rigor, and overall quality of our work.

In response to the latest round of feedback, we have carefully addressed all the points raised, and detailed modifications have been made throughout the manuscript. All changes in this second revision are highlighted in red for your convenience. Our point-by-point responses to each comment are provided below.

We are deeply thankful for the constructive and thoughtful suggestions throughout the review process, which have significantly improved the quality of our paper. We hope that the current version meets with your approval and look forward to your response.

Sincerely,

Shuangshuang Le

**Reviewer #3:**

**Comment 1**

The authors addressed the reviewer’s comment. However, they should clarify which shRNAs against LAMP2A were used. Based on Figure 3B, LV-shL2A-2 caused maximum knockdown. While the authors did not indicate which LV-sh-L2A was used in Fig. 4 and Fig. 6, it is likely that LV-shL2A-2 was used since other clones did not cause complete knockdown. While the authors indicated that they used a second independent shRNA in supplementary Fig.2A, B and C to rule out off-target effects but according to Fig.S2 A, B and C, they repeated the experiment with the same clone and that’s why the results are very similar. They should clearly specify which shRNA was used and how the levels of shRNA affected the results. In addition, they should perform the experiments with 2 distinct shRNAs.

**Response:**

We sincerely thank the reviewer for this important comment and for highlighting the need for greater clarity and rigor in our shRNA experiments. We apologize for the lack of clarity in our initial description. Below, we provide a point-by-point response.

**1. Clarification of shRNA used in the main figures:**

The reviewer is correct that LV-shL2A-2 showed the strongest knockdown efficiency in Figure 3B. Indeed, all functional experiments in Figures 4 and 6 were performed using LV-shL2A-2. We have now explicitly stated this in the revised Results (Section 3.3) and updated the corresponding figure legends for clarity.

**2. Experiment with a second independent shRNA:**

We agree that validating key findings with a second, distinct shRNA is essential to confirm the specificity of the observed phenotypes. In response, we have performed additional experiments using an independent LAMP2A-targeting shRNA (LV-shL2A-3).

These new data are presented in the Supplementary Figure S2 and confirm that knockdown with LV-shL2A-3 similarly sensitizes MKN45 cells to H₂O₂-induced apoptosis, replicating the core phenotypes observed with LV-shL2A-2. We have updated the Results section (3.4) and Discussion to reference this confirming data, strengthening the conclusion that the effects are due to LAMP2A knockdown and not off-target artifacts.

We believe these revisions have significantly strengthened the reliability of our genetic knockdown data.

**Reviewer #5:**

**Comment 1**

**-**The abstract would benefit from improved narrative flow. Currently, it reads as a list of experimental steps rather than a cohesive summary. Methodological details (e.g., specific assays used) can be reduced in favor of emphasizing key findings and conclusions.
- Revise the introduction to improve grammar and flow. Redundant or closely related sentences should be merged for clarity (e.g., the definition of oxidative stress and ROS accumulation).
- Improve sentence flow and use clearer transitions when introducing CMA and the role of LAMP2A (e.g., “CMA requires lysosomal-associated membrane protein 2A (LAMP2A) for substrate translocation”).
- Revise awkward or overly long sentences for clarity. For example, the sentence describing LAMP2A downregulation in HCC should be restructured to improve readability and logical flow.
- Correct grammatical errors such as the missing verb in the sentence:
“Consistent with previous reports [14], we confirmed that LAMP2A shows consistently higher expression across gastric adenocarcinoma tissues and cell lines.”
- The statement implying that elevated LAMP2A levels directly indicate enhanced CMA activity is not sufficiently supported by the data. Please revise to a more cautious interpretation (e.g., “may indicate increased CMA activity”).

**Response:**

We are very grateful to the reviewer for these detailed and constructive suggestions regarding the language, clarity, and interpretation in our manuscript. We have thoroughly revised the text accordingly.

1. **Abstract:**

We have rewritten the abstract to improve its narrative flow, reducing methodological details and emphasizing the key findings and conclusions. The revised version now more clearly outlines the study's motivation, central discovery (hyperoxidized DJ-1 as a novel CMA substrate), and its biological implication.

“Chaperone-mediated autophagy (CMA) promotes cancer cell survival by selectively removing oxidatively damaged proteins, yet its precise molecular mechanisms and role in redox adaptation remain incompletely understood. This study aimed to elucidate the function of CMA in regulating oxidative stress resistance in gastric cancer (GC) cells, focusing on the LAMP2A–DJ-1 regulatory axis. LAMP2A expression was assessed in GC tissues and cell lines via immunohistochemistry, qPCR, and western blot. Oxidative stress models were established using hydrogen peroxide (H₂O₂). Genetic manipulation of LAMP2A was performed to evaluate its impact on cell proliferation, apoptosis, and CMA substrate recognition. Protein interactions were examined by co-immunoprecipitation and immunofluorescence. LAMP2A was upregulated in GC and further induced by oxidative stress. Knockdown of LAMP2A impaired CMA activity, sensitizing GC cells to H₂O₂-induced apoptosis. DJ-1, an antioxidant protein, was identified as a CMA substrate containing a conserved KFERQ-like motif. Oxidative stress enhanced DJ-1–LAMP2A interaction and promoted their lysosomal colocalization. LAMP2A deficiency led to accumulation of hyperoxidized DJ-1, concomitant with upregulation of pro-apoptotic BAX and downregulation of anti-apoptotic Bcl-2. We identify hyperoxidized DJ-1 as a novel CMA substrate and demonstrate that LAMP2A-dependent clearance of oxidized DJ-1 constitutes a key adaptive mechanism that maintains redox homeostasis and promotes survival in gastric cancer cells under oxidative stress.”

2. **Introduction:**

We have merged redundant sentences and improved grammatical flow, particularly in the paragraphs defining oxidative stress and introducing CMA.

The description of LAMP2A's role has been rephrased for clarity: “CMA requires the lysosomal membrane receptor LAMP2A for substrate translocation.”

The sentence regarding LAMP2A in HCC has been restructured for better readability and logical connection.

In summary, we have rewritten the Introduction section:

Gastric cancer (GC) ranks fifth in global incidence and third in cancer-related mortality [1]. Although biomarkers such as PD-L1, MSI, and HER2 can guide treatment decisions [2], their limited specificity and sensitivity underscore the urgent need to discover novel molecular markers, which is essential for improving early diagnosis and patient survival. Oxidativestress, characterized by an imbalance between reactive oxygen species (ROS) production and antioxidant defenses plays a dual role in tumor biology [3]. While moderate ROS levels can drive proliferation and survival, excessive accumulation induces cytotoxic damage [4]. The rapid proliferation of tumor cells generates high levels of ROS; however, they can evade senescence and apoptosis by enhancing their intrinsic antioxidant defenses [5]. Autophagy, a conserved lysosomal degradation pathway, is crucial for maintaining cellular homeostasis under stress. Among its three forms—macroautophagy, microautophagy, and chaperone-mediated autophagy (CMA)—CMA stands out for its selectivity [6]. CMA targets cytosolic proteins bearing KFERQ-like motifs, which are recognized by heat shock cognate 70 kDa protein (HSC70) and translocated into lysosomes via the receptor lysosome-associated membrane protein 2A (LAMP2A) for degradation [7,8]. CMA activation has been implicated in promoting tumor progression across various malignancies [9]. For example, in hepatocellular carcinoma, LAMP2A downregulation promotes proliferation and migration via YAP1- and IL6ST-dependent pathways [10]. Conversely, in glioma models, increased CMA activity—achieved through LAMP2A upregulation—similarly enhances proliferation and invasion [11]. The functional importance of CMA in oncogenesis is further underscored by studies in non-small cell lung cancer (NSCLC), where inhibiting the HSC70–LAMP2A interaction blocks CMA and suppresses tumor growth [12]. In breast cancer, CMA promotes cell survival by selectively degrading oxidatively damaged proteins [13], highlighting its critical role in oxidative stress response. Notably, in the context of gastric cancer, LAMP2A—the key receptor in CMA—has been identified not only as a potential early biomarker for gastric mucosal precancerous lesions but also as a specific marker for gastric cancer itself [14]. Therefore, our study will further explore the role and molecular mechanism of CMA in regulating the anti-oxidative stress of gastric cancer cells. We hope establish a mechanistic framework for CMA-mediated oxidative stress adaptation in gastric cancer cells.

3. **Results Section (3.1):**

The grammatical error has been corrected: “Consistent with previous reports [14], we confirmed that LAMP2A is consistently upregulated in gastric adenocarcinoma tissues and cell lines.”

Following the reviewer’s advice, we have tempered our interpretation regarding CMA activity. The sentence now reads:“Given that LAMP2A is the rate-limiting factor of CMA, its elevated level may indicate enhanced CMA activity in gastric cancer cells compared to normal gastric mucosa cells.”

4. **General Language Polish:**

We have carefully reviewed the entire manuscript to revise awkward or overly long sentences, ensure consistent use of technical terms, and improve transitional phrases for a smoother reading experience. Please refer to the Revised Manuscript with Track Changes for specific modifications.
