# Supplementary material for: LAMP2A-dependent chaperone-mediated autophagy enhances oxidative stress resistance in gastric cancer cells through selective degradation of accumulated oxidized DJ-1
Source: PLoS One. 2026 May 15;21(5):e0331823. doi: 10.1371/journal.pone.0331823 (PMC13178873; doi:10.1371/journal.pone.0331823)
Supplement: Attachment — Submitted filename: Response to Reviewers .docx [file pone.0331823.s008.docx]

Dear Editor,

Thank you again for your careful review of our manuscript and for raising the important concern regarding the molecular weight labels in our original Figure 6A.

We fully acknowledge that the labels in the original figure did not correspond to the actual ladder positions. This was an error on our part, and we sincerely apologize for any confusion it may have caused.

**Original mistake:**

In the original submission, we labeled the bands with theoretical molecular weights (Bcl‑2 as 26 kDa, DJ‑1 as 20 kDa) without referring to the ladder positions on the blot. We now understand that the correct practice is to label bands strictly according to the observed ladder, and any deviation from theoretical values should be addressed in the figure legend or text. We regret this oversight.

**Regarding the abnormal migration in the original blot:**

In the original experiment, Bcl‑2 appeared at approximately 20 kDa (expected 26 kDa) and DJ‑1 at approximately 25 kDa (expected 20 kDa). After re‑examining our records and experimental conditions, we suspect that this unusual migration was caused by technical issues specific to that run (e.g., marker batch variation, gel polymerization, or electrophoresis conditions).

**Corrective action taken:**

We have repeated the Western blot experiments under optimized and carefully controlled conditions (fresh molecular weight markers, proper gel preparation, and standard electrophoresis protocols). The new blots show that both Bcl‑2 and DJ‑1 migrate at positions consistent with their theoretical molecular weights, with only minor deviations within 2–3 kDa—which is routinely acceptable for Western blot analysis. Representative images from these repeated experiments are provided in the revised Figure 6A. We have also uploaded the raw, uncropped Western blot data for the repeated experiments as supporting information.

We hope to replace the original Figure 6A with the new, reliable data. All other figures and conclusions in the manuscript remain unchanged. We apologize again for any confusion caused by the original figure and thank you for the opportunity to correct it.

Figure 6A

Sincerely,

Pang maogui

On behalf of all authors
